# Metabolic and Hepatic Profiles of Non-Obese and Obese Metabolic Dysfunction-Associated Steatotic Liver Disease in Adolescents: The Role of FibroScan Parameters,Fibroblast Growth Factor-21, and Cytokeratin-18

**DOI:** 10.5152/tjg.2025.24760

**Published:** 2025-02-03

**Authors:** Murat Keskin, Hanife Aysegul Arsoy, Ozlem Kara, Emre Sarandol, Aylin Beyaz, Nizameddin Koca, Yusuf Yilmaz

**Affiliations:** 1 Department of Gastroenterology and Hepatology, KTO Karatay University Faculty of Medicine, Konya, Türkiye; 2 Department of Pediatric Gastroenterology, Hepatology and Nutrition, University of Health Sciences Bursa, Yüksek İhtisas Training and Research Hospital, Bursa, Türkiye; 3 Department of Pediatric Endocrinology, University of Health Sciences Bursa, Yüksek İhtisas Training and Research Hospital, Bursa, Türkiye; 4 Department of Biochemistry, Bursa Uludağ University, Bursa, Türkiye; 5 Department of Internal Medicine, University of Health Sciences Bursa Faculty of Medicine, Bursa City Training and Research Hospital, Bursa, Türkiye; 6 Department of Gastroenterology and Hepatology, Riza Recep Tayyip Erdoğan University, Rize, Türkiye

**Keywords:** Metabolic dysfunction-associated steatotic liver disease, adolescents, fibroblast growth factor-21, cytokeratin-18, FibroScan, biomarkers

## Abstract

**Background/Aims:**

Metabolic dysfunction-associated steatotic liver disease (MASLD) in adolescents, including non-obese phenotypes, is an increasingly important public health issue. The current study investigated the use of controlled attenuation parameter (CAP) and liver stiffness measurement (LSM) as non-invasive tools, along with fibroblast growth factor-21 (FGF-21) and cytokeratin-18 (CK-18), in non-obese MASLD, obese MASLD, and healthy control groups, exploring metabolic and hepatic profiles in these groups.

**Materials and Methods:**

This cross-sectional study recruited 195 adolescents aged 9-18 years, stratified into controls (n = 92), non-obese MASLD (n = 32), and obese MASLD (n = 39) groups according to FibroScan and MASLD diagnostic criteria. FibroScan measured LSM and CAP, while enzyme-linked immunosorbent assay kit (ELISA) was used to analyze serum FGF-21 and CK-18 levels. Anthropometric, metabolic, and liver enzyme parameters were assessed.

**Results:**

: Metabolic dysfunction-associated steatotic liver disease groups had higher LSM than controls. Fibroblast growth factor-21 levels were significantly higher in MASLD groups, especially in obese MASLD, while CK-18 levels showed variability without significant group differences. Obese MASLD adolescents had marked metabolic dysfunction with higher insulin, homeostasis model assessment for insulin resistance, triglycerides, and liver enzymes compared to non-obese MASLD and controls.

**Conclusion:**

Fibroblast growth factor-21 has emerged as a potential biomarker for assessing metabolic dysfunction in MASLD, while LSMs from FibroScan provide valuable insights into fibrosis risk. Elevated FGF-21 levels and FibroScan parameters reflect their potential usefulness in non-invasive assessment of MASLD severity, particularly in obese adolescents. However, further longitudinal studies are needed to establish their roles in predicting disease progression and guiding clinical management.

**Main Points**

- Liver stiffness measurement from FibroScan was significantly higher in MASLD groups than controls, reflecting its utility in assessing liver fibrosis in adolescents with MASLD.
- Elevated FGF-21 levels in MASLD groups, particularly in obese MASLD, indicate its potential as a biomarker for metabolic dysfunction, reflecting disease severity.
- Cytokeratin-18 levels were higher in MASLD groups but did not reach statistical significance, indicating variability in its utility for early-stage MASLD assessment.
- Non-obese MASLD adolescents exhibit significant metabolic and hepatic abnormalities, indicating that MASLD can occur even with modest adiposity, independent of obesity.
- The study highlights the potential role of non-invasive tools like LSM and FGF-21 in assessing MASLD severity in adolescents.

## Introduction

Metabolic dysfunction-associated steatotic liver disease (MASLD), previously known as non-alcoholic fatty liver disease (NAFLD), has emerged as the most prevalent chronic liver disease in adolescents, paralleling the rising rates of pediatric obesity and metabolic syndrome. Current estimates suggest that approximately 25% of children and adolescents worldwide are affected, with obesity increasing prevalence to over 50%.^1^ Metabolic dysfunction-associated steatotic liver disease is not limited to obesity; the non-obese variant is becoming more recognized as a distinct phenotype with specific metabolic and hepatic features, presenting challenges in diagnosis and management.^2^ This dual spectrum highlights the necessity of comprehending MASLD across various phenotypes, particularly in non-obese populations where disease presentation is frequently overlooked.

Non-invasive diagnostic biomarkers and tools are crucial for evaluating the severity and progression of MASLD, particularly when a liver biopsy is impracticable in a population such as adolescent populations. FibroScan is a widely used non-invasive imaging tool to measure liver stiffness (liver stiffness measurement [LSM]) and hepatic fat content (controlled attenuation parameter [CAP]).^3^ This study assessed liver fat accumulation using the CAP, while LSM was used to evaluate fibrosis. Controlled attenuation parameter was utilized as a group-defining criterion, whereas LSM served as an outcome measure to assess liver fibrosis in MASLD patients.

In addition to imaging tools, emerging biomarkers like fibroblast growth factor-21 (FGF-21) and cytokeratin-18 (CK-18) have shown potential to reflect metabolic dysfunction and liver injury, particularly in MASLD. Fibroblast growth factor-21, a hepatokine crucial for glucose and lipid metabolism regulation, increases in response to metabolic stress in MASLD, with higher levels correlating with obesity, insulin resistance, and disease severity.^4^ Cytokeratin-18, a hepatocyte cytoskeletal protein, is released into the bloodstream during apoptosis, particularly as caspase-cleaved CK-18 (ccCK-18). Elevated CK-18 levels reflect hepatocyte apoptosis and are associated with MASLD severity.^5^

These are advances, however, that need to be taken further into the interplay of FibroScan parameters, FGF-21, CK-18, and MASLD phenotypes, particularly in adolescents. This study combines non-invasive imaging with biomarker analyses to comprehensively investigate MASLD in non-obese and obese adolescents vs. controls. It is now necessary to understand these relations for refinement into diagnostic algorithms and targeted interventions involving the broader implications in pediatric outcomes. The non-obese MASLD phenotype presents unique challenges in diagnosis, as it often occurs in individuals without significant adiposity. Understanding this phenotype is crucial for developing appropriate diagnostic and therapeutic approaches. There is limited understanding of the non-obese MASLD phenotype in adolescents, a condition often overlooked in clinical practice. Furthermore, while FibroScan parameters and biomarkers like FGF-21 and CK-18 have shown promise in adults, their utility in pediatric populations remains unclear. This study aims to address these gaps by integrating FibroScan parameters and biomarker analyses to explore metabolic and hepatic profiles across MASLD phenotypes in adolescents.

## Materials and Methods

This cross-sectional observational study aimed to evaluate clinical, laboratory, and imaging parameters among adolescents with non-obese MASLD, obese MASLD, and controls. The study was approved by the Ethics Committee of Karatay University Faculty of Medicine (13.07.2021; 2021/19) and conducted following the Declaration of Helsinki. Written informed consent was obtained from participants and/or their legal guardians.

### Selection of Patients and Criteria

A total of 195 adolescents aged 9-18 years were enrolled. Hepatic steatosis was diagnosed using FibroScan with a CAP cutoff of 225 dB/m.^6^ Ninety-two adolescents without hepatic steatosis were included as controls. Among 103 adolescents with hepatic steatosis, 39 were obese (body mass index [BMI] ≥95th percentile for age and sex) and met the MASLD criteria,^7^ forming the “obese MASLD” group. The remaining 54 non-obese adolescents with hepatic steatosis were further evaluated, and 22 who did not meet the MASLD criteria^7^ were excluded. The remaining 32 participants constituted the “non-obese MASLD” group.

### Inclusion Criteria

- Adolescents aged 9-18 years.
- MASLD groups: hepatic steatosis was confirmed by FibroScan, and metabolic risk factors were present based on established diagnostic criteria.^7^
- Control group: no evidence of hepatosteatosis, metabolic dysfunction, or liver-related conditions.

### Exclusion Criteria

- History of chronic liver diseases unrelated to MASLD (e.g., viral hepatitis, autoimmune hepatitis, Wilson’s disease, and alpha-1 antitrypsin deficiency).
- Secondary factors contributing to hepatosteatosis (e.g., medication use, alcohol consumption, and malnutrition).
- Secondary causes of obesity, including endocrinological disorders such as Cushing syndrome, hypothyroidism, acromegaly, or Polycyctic ovary syndrome.
- Clinical features suggestive of endocrinological disorders (e.g., rapid unexplained weight gain, moon facies, and hirsutism).
- Inability to provide informed consent.

### Clinical and Laboratory Assessment

Anthropometric measurements included height, weight, BMI, and waist circumference (WC). Blood pressure (systolic and diastolic) was measured seated after a 5-minute rest. Laboratory parameters included fasting blood glucose, insulin, homeostasis model assessment for insulin resistance (HOMA-IR), complete blood count (CBC), lipid profile (i.e., total cholesterol, triglycerides [TGs], high-density lipoprotein, and low-density lipoprotein [LDL]), liver enzymes (i.e., aspartate transaminase [AST], alanine transaminase, gamma-glutamyl transferase, and alkaline phosphatase), thyroid function (i.e., thyroid-stimulating hormone and free thyroxine), vitamin D, calcium, phosphorus, and uric acid.

Blood samples were collected from the antecubital vein in EDTA and non-additive tubes, centrifuged at 1500 *g* for 10 minutes, and processed immediately. Serum for CK-18 and FGF-21 were measured in duplicate using enzyme-linked immunosorbent assay (ELISA) kits (ELK Biotechnology Lab, Wuhan, China) on serum aliquots stored at −80°C until analysis. Routine biochemical tests were performed using an Aeroset System autoanalyzer, and CBC was measured using the Abbott Cell-Dyn 3700SL.

### Biomarker Analysis: Fibroblast Growth Factor-21 and Cytokeratin-18 

Two biomarkers relevant to MASLD pathophysiology, FGF-21 and CK-18, were analyzed. Serum FGF-21 levels were measured using an ELISA kit, following the manufacturer’s instructions. Cytokeratin-18 levels were also measured using a specific ELISA kit and expressed in U/L.

### FibroScan Imaging and Liver Stiffness Measurement

Fatty liver and liver stiffness were assessed using FibroScan, performed by a certified physician to ensure accuracy. Hepatic fat content was quantified using the CAP in decibels per meter (dB/m), while fibrosis was assessed via LSM in kilopascals (kPa). Hepatosteatosis was defined by a CAP cutoff of 225 dB/m.^6^ Reliable measurements required at least 10 valid readings with an interquartile range below 30% of the median. Higher LSM values indicate increased liver stiffness and fibrosis severity.

### Statistical Analysis

All statistical analyses were performed in SPSS, version 27 (IBM Corp., Armonk, NY, USA). Continuous variables were summarized as mean ± SD, while categorical variables were presented as frequency and percentage. The normality test for the data was examined by using the Shapiro–Wilk test. Comparisons among the groups in continuous variables were analyzed by 1-way ANOVA or the Kruskal–Wallis test accordingly with a post hoc Bonferroni adjustment in pairwise comparisons.

Categorical variables were compared using chi-square or Fisher’s exact test. The differences were considered significant when the *P*-value was less than .05. The risk factors of MASLD were investigated in adolescents recruited from 3 groups:

P1: Control vs. non-obese MASLD.
P2: Control vs. obese MASLD.
P3: non-obese vs. obese MASLD.

## Results

### Demographic and Anthropometric Characteristics

Age, gender, and puberty status were similar across groups (*P* > .05). In contrast, weight was significantly higher in MASLD groups (50.93 ± 11.79, 70.33 ± 13.21, and 94.5 ± 17.51 kg; *P < .*001), with significant pairwise differences between controls and MASLD groups (*P < .*001) and between non-obese and obese MASLD (*P < .*001). Body mass index also differed significantly across groups (19.91 ± 2.71, 27.26 ± 2.35, and 35.03 ± 4.15 kg/m^2^; *P < .*001), with all pairwise comparisons significant (*P < .*001) (Table 1).

Systolic blood pressure was significantly higher in MASLD groups (109.84 ± 8.91, 118.13 ± 8.87, and 120.13 ± 13.64 mm Hg; *P < .*001), with significant differences between controls and both MASLD groups (*P < .*001) but not between non-obese and obese MASLD (*P = .*976). Diastolic blood pressure also differed significantly (69.18 ± 7.78, 76.88 ± 6.32, and 75.38 ± 11.49 mm Hg; *P < .*001), with significant differences for controls vs. non-obese (*P < .*001) and obese MASLD (*P = .*006) but not between MASLD groups (*P = .*514). Waist circumference was significantly higher in MASLD groups (70.13 ± 7.75, 94.06 ± 9.45, and 111.6 ± 13.07 cm; *P < .*001), with all pairwise comparisons significant (*P < .*001) (Table 1).

### Clinical Features

The prevalence of acanthosis nigricans was significantly higher in MASLD groups (3.3%, 25%, and 59%; *P < .*001), with significant pairwise differences across all groups (*P < .*001). Similarly, metabolic syndrome prevalence differed significantly (1.1%, 46.9%, and 69.2%; *P < .*001), with significant pairwise differences between all groups (control vs. non-obese MASLD, *P < .*001; control vs. obese MASLD, *P < .*001; non-obese vs. obese MASLD, *P = .*001).

### Laboratory Parameters

Fasting blood glucoase levels did not differ significantly across groups (86.2 ± 7.89, 88.41 ± 7.24, and 87.15 ± 7.38 mg/dL;*P = .*359). However, insulin levels were significantly higher in MASLD groups (12.41 ± 6.03, 28.71 ± 16.1, and 37.84 ± 22.26 mIU/L; *P < .*001), with significant pairwise differences between controls and both MASLD groups (*P < .*001) and between non-obese and obese MASLD (*P = .*014). HOMA-IR levels were also significantly elevated in MASLD groups (2.67 ± 1.42, 6.31 ± 3.85, and 8.38 ± 5.33; *P < .*001), with significant pairwise differences across all groups (Table 2).

Aspartate transaminase (AST) levels were significantly higher in MASLD groups (17.21 ± 4.15, 24.49 ± 14.78, and 28.05 ± 14.52 IU/L; *P < .*001), with significant pairwise differences between controls and both MASLD groups. Alanine transaminase (ALT) levels differed significantly (12.99 ± 7.05, 42.19 ± 42.8, and 45.51 ± 35.41 IU/L; *P < .*001), with similar pairwise differences. Gamma-glutamyl transferase was elevated in MASLD groups (11.13 ± 4.82, 23.22 ± 17.25, and 24.95 ± 13.87 IU/L; *P < .*001), with significant differences between controls and MASLD groups but not between non-obese and obese MASLD (Table 2).

Total cholesterol levels were significantly higher in MASLD groups compared to controls (143.04 ± 28.25, 164.38 ± 35.96, and 160.9 ± 26.83 mg/dL; *P < .*001), with pairwise differences significant for control versus both MASLD groups but not between MASLD groups. Triglycerides were also elevated in MASLD groups (79.21 ± 29.61, 152.75 ± 66.74, and 153.41 ± 60.92 mg/dL; *P < .*001), with significant pairwise differences except between MASLD groups. Low-density lipoprotein levels differed significantly (75.41 ± 22.82, 87.39 ± 28.57, and 87.8 ± 26.16 mg/dL; *P = .*015), with control differing from both MASLD groups. High-density lipoprotein was significantly reduced in MASLD groups (54.76 ± 13.13, 45.91 ± 11.41, and 41.99 ± 8.64 mg/dL; *P < .*001), with significant differences between control and MASLD groups (Table 2).

Thyroid-stimulating hormone levels varied significantly across groups (2.28 ± 1.12, 3.08 ± 1.59, and 2.82 ± 1.48 mIU/L; *P = .*014), with significant differences between control and non-obese MASLD. Free thyroxine also differed significantly (1.22 ± 0.2, 1.16 ± 0.14, and 1.14 ± 0.17 ng/dL; *P = .*037), with a significant difference between control and obese MASLD. While vitamin D levels showed no significant differences, calcium levels were higher in MASLD groups (9.38 ± 0.45, 9.63 ± 0.41, and 9.56 ± 0.41 mg/dL; *P = .*008), with pairwise differences significant for controls versus both MASLD groups. Uric acid levels were significantly elevated in MASLD groups (4.03 ± 0.95, 5.33 ± 1.74, and 5.78 ± 1.64 mg/dL; *P < .*001), with pairwise differences significant between controls and MASLD groups (Table 2).

### Liver Stiffness and Fat Accumulation

This study utilized the CAP as a criterion to classify participants into MASLD and control groups. Therefore, higher CAP values in MASLD groups were expected and should not be interpreted as a novel finding. Controlled attenuation parameter values, reflecting hepatic fat content, were significantly higher in MASLD groups than in controls (187.86 ± 23.98 vs. 283.03 ± 39.48 vs. 299.26 ± 47.45 dB/m, *P < .*001). The LSM, evaluated as an outcome measure for fibrosis, was also significantly elevated in MASLD groups, indicating increased fibrosis risk (control: 4.15 ± 0.68 kPa; non-obese MASLD: 5.15 ± 1.00 kPa; obese MASLD: 6.03 ± 1.79 kPa, *P < .*001). Pairwise comparisons showed that LSM values were significantly different across all groups (Table 3).

### Biomarkers: Fibroblast Growth Factor-21 and Cytokeratin-18 

Fibroblast growth factor-21 levels were significantly higher in MASLD groups compared to controls (control: 7.31 ± 15.96 pg/mL; non-obese MASLD: 14.76 ± 24.74 pg/mL; obese MASLD: 20.65 ± 36.1 pg/mL; *P = .*002). Pairwise comparisons showed higher FGF-21 levels in non-obese MASLD vs. controls (*P = .*031) and in obese MASLD vs. controls (*P = .*001), but no significant difference between non-obese and obese MASLD groups (*P = .*38). The elevated FGF-21 levels in MASLD groups, regardless of obesity status, indicate its potential as a marker of metabolic stress in adolescents. However, its role in distinguishing MASLD severity and progression requires further investigation through longitudinal studies.

Cytokeratin-18 levels were higher in MASLD groups than controls (0.64 ± 0.77 U/L, 0.78 ± 0.74 U/L, and 1.34 ± 2.77 U/L, respectively; *P = .*127). However, CK-18 did not reach statistical significance in any pairwise comparison, reflecting variability in hepatocyte apoptosis across the population and suggesting the limited utility of CK-18 in early-stage MASLD assessment.

## Discussion

This study is among the first to evaluate LSM and CAP alongside biomarkers such as FGF-21 and CK-18 in adolescents with MASLD. Our findings provide insights into the metabolic and hepatic profiles of non-obese and obese MASLD phenotypes, emphasizing the importance of non-invasive tools in pediatric liver disease assessment. The findings offer valuable insights into the non-obese MASLD phenotype, challenging the traditional association of MASLD with obesity alone.

Our findings reveal higher LSM values in both MASLD groups, with the highest levels in obese MASLD, highlighting obesity’s impact on fibrosis severity. Elevated FGF-21 levels in MASLD groups, especially in obese MASLD, suggest its potential as a marker of metabolic dysfunction. In contrast, CK-18 levels, while higher, lacked statistical significance, likely reflecting variability in early-stage disease.

Obese MASLD adolescents also exhibited significantly higher BMI, WC, TGs, LDL, insulin, HOMA-IR, and AST/ALT levels compared to non-obese MASLD and controls, underscoring the metabolic burden of obesity. Higher BMI and WC, both significant demographic differences, reflect visceral adiposity and metabolic dysregulation, key drivers of hepatic steatosis and fibrosis.^1^ These findings reinforce the multifaceted nature of MASLD pathophysiology and emphasize the need for early, targeted interventions to address this growing concern in adolescents.^3^

The control group in the present study also showed a significantly lower mean weight and BMI compared with both MASLD groups. This finding agrees with previous literature indicating that higher body weight and BMI are predictors of MASLD severity, especially in adolescents.^8^ Increased WC among MASLD adolescents has also been associated with greater visceral fat accumulation, which directly contributes to insulin resistance and hepatic lipid accumulation—both implicated in MASLD pathogenesis.^9^

Of note, although the MASLD non-obese group exhibited a significantly lower BMI and WC when compared with the obese MASLD group, it is higher compared to control subjects.^2^ This finding confirms that modest increases in adiposity with the non-obese range are associated with metabolic dysfunction sufficient to drive hepatic steatosis, as reported in several lean MASLD phenotype studies. The findings in non-obese MASLD highlight this phenotype as a distinct entity, with modest increases in adiposity sufficient to drive hepatic steatosis. These results align with studies identifying lean MASLD as a heterogeneous condition driven by metabolic dysregulation rather than excess body weight alone.^2^

Besides, the high values of BMI and WC are directly related to the severities in the outcome of liver function in MASLD patients, such as an increased risk for the degree of fibrosis. Recent studies using non-invasive imaging techniques like FibroScan have confirmed that anthropometric metrics of increased adiposity are related to higher liver stiffness measurements in MASLD adolescents.^10^ The mentioned correlation represents the need for the regular performance of anthropometric and imaging examinations in adolescents at risk for MASLD for the detection of early signs of the disease process.

These laboratory differences highlight the metabolic and hepatic dysfunction in adolescents with MASLD. Both MASLD groups showed higher levels of TGs, LDL, insulin, HOMA-IR, and AST/ALT compared to controls, with the obese MASLD group having the highest values. This supports the established role of dyslipidemia and insulin resistance in MASLD pathogenesis.^1^

The metabolic burden of MASLD, evident in elevated TGs, LDL, HOMA-IR, and transaminase levels, highlights the role of dyslipidemia and insulin resistance in disease pathogenesis. The highest values in obese MASLD suggest a more severe phenotype requiring targeted metabolic interventions.^3,11^ The substantial LDL elevations emphasize MASLD’s association with an atherogenic lipid profile, indicating increased cardiovascular risk in obese adolescents.^12^

Insulin and HOMA-IR levels were significantly higher in MASLD groups compared to controls, with the highest levels in obese MASLD. Insulin resistance is a key driver of MASLD, promoting hepatic steatosis through increased free fatty acid flux to the liver, enhanced lipogenesis, and suppressed fatty acid oxidation.^13^ These findings align with evidence showing elevated insulin resistance in both obese and non-obese MASLD patients.^2^

Liver stiffness measurement values were significantly higher in both MASLD groups than controls, with the highest increase observed in obese MASLD. Elevated LSM reflects fibrosis, a hallmark of MASLD progression, consistent with previous studies supported by histologic evidence of fibrosis, even in pediatric populations.^3^ The stepwise increase in LSM from controls to non-obese and obese MASLD suggests an additive effect of obesity on liver stiffness, likely driven by metabolic dysfunction and chronic low-grade inflammation.^14^ Liver stiffness measurement is a validated marker for fibrosis staging and progression in MASLD and is widely used for non-invasive monitoring. Recent guidelines emphasize Fibroscan’s utility in detecting fibrosis, particularly in adolescents with obesity and metabolic risk.^1^ The strong association observed in this study between severe MASLD and elevated LSM highlights its crucial role as a diagnostic tool, aiding clinicians in identifying advanced metabolic dysfunction and informing future management strategies.

In this study, the CAP was utilized as a criterion to classify participants into MASLD and control groups, reflecting hepatic fat content. Therefore, higher CAP values in MASLD groups were expected and should not be interpreted as a novel finding. Controlled attenuation parameter values were significantly higher in obese MASLD adolescents, indicating a greater burden of hepatic steatosis. This finding aligns with the established role of obesity in promoting liver fat accumulation through increased lipogenesis and adipocyte-derived free fatty acid flux to the liver.^15,16^ Interestingly, although non-obese MASLD patients had lower CAP values than obese MASLD patients, their CAP levels were significantly higher than those of controls, suggesting that hepatic fat accumulation is not limited to obesity but also occurs in lean individuals with metabolic dysregulation. These results underscore that non-obese MASLD represents a distinct phenotype driven by metabolic dysfunction rather than excess adiposity alone.^2^

This study highlights the diagnostic potential of FGF-21 in adolescents with MASLD, particularly for understanding metabolic and liver dysfunction in pediatric populations. Fibroblast growth factor-21 levels were significantly higher in MASLD groups, with the highest levels observed in obese MASLD patients, aligning with its role as a hepatokine secreted in response to metabolic stress, including insulin resistance, lipid metabolism dysregulation, and hepatic steatosis. Fibroblast growth factor-21 promotes lipid oxidation, glucose regulation, and thermogenesis as a compensatory mechanism.^4^ However, while its association with hepatic TGs and fibrosis has been reported, further longitudinal studies are necessary to validate its predictive value and refine its clinical utility in pediatric populations.^16,17^

Studies have shown that FGF-21 strongly correlates with hepatic triglyceride content and MASLD histologic features, including inflammation and fibrosis.^17^ This supports our findings, where obese MASLD adolescents exhibited the highest levels, likely due to more significant hepatic lipid accumulation and metabolic stress. Additionally, FGF-21 has been proposed as a non-invasive biomarker for distinguishing MASLD from healthy controls, with levels correlating with liver stiffness.^16^ These findings underscore FGF-21’s potential as a valuable marker for assessing disease severity, particularly in obesity-driven MASLD. However, the cross-sectional design of this study limits the ability to establish causality or predict disease progression. While FGF-21 holds potential for MASLD stratification, further longitudinal research with larger cohorts is necessary to validate its clinical utility and refine its application in pediatric populations.

Cytokeratin-18 levels were elevated in MASLD groups but did not reach statistical significance, likely reflecting early-stage disease in this cohort. As a marker of hepatocyte apoptosis, CK-18 is more sensitive in advanced disease stages. The variability in CK-18 levels may also result from disease heterogeneity and the overlap between apoptosis and other liver injury mechanisms, such as necrosis and metabolic stress. Recent studies suggest that combining CK-18 with other markers, like FGF-21, could enhance diagnostic accuracy, particularly in identifying MASLD phenotypes and assessing the risk of progression to NASH.^5,18,19^ Although CK-18 levels were higher in MASLD groups, the lack of statistical significance may reflect the early-stage nature of the disease in this cohort or variability in the biomarker’s sensitivity for detecting minimal fibrosis. Further studies should explore CK-18’s role in combination with other biomarkers to improve diagnostic accuracy.

This study demonstrates the utility of FibroScan parameters (LSM and CAP) and biomarkers like FGF-21 in distinguishing MASLD phenotypes in adolescents. Elevated LSM values in both MASLD groups reflect increased liver stiffness and fibrosis risk, with the highest levels observed in obese MASLD, suggesting an additive impact of obesity on fibrosis severity. Higher CAP values emphasize FibroScan’s ability to detect hepatic fat accumulation. However, these findings should be interpreted cautiously, given the reliance on predefined CAP and LSM thresholds, which may vary across studies. Future longitudinal studies incorporating liver biopsy validation are necessary to establish standardized thresholds and improve diagnostic accuracy in pediatric populations.

Significantly higher FGF-21 levels in MASLD groups support its role as a marker of metabolic dysfunction, with the highest levels in obese MASLD indicating its utility in advanced disease. Cytokeratin-18 levels, though elevated in MASLD groups, lacked statistical significance, reflecting variability in its role as a marker of hepatic injury in early MASLD. These findings support using FibroScan, FGF-21, and CK-18 as non-invasive tools for assessing MASLD severity. However, to confirm their predictive value, longitudinal studies are needed to validate these biomarkers across diverse pediatric populations.

### Limitations

- The cross-sectional design limits the ability to infer causality and assess MASLD progression over time.
- The lack of liver biopsy validation, the gold standard for MASLD diagnosis, may reduce the precision of FibroScan-based fibrosis staging, particularly in pediatric populations. Ethical and practical challenges in obtaining biopsies necessitated reliance on non-invasive methods like Fibroscan, which requires further refinement through histological validation in future studies.
- The small sample size for non-obese MASLD may have limited the power to detect subtle biomarker differences, such as CK-18 variability in early-stage disease.
- Reliance on predefined CAP and LSM thresholds, which vary across studies, highlights the need for standardized diagnostic criteria for pediatric populations.
- The study focused exclusively on adolescents, limiting the generalizability of findings to younger children or adults.

This study highlights the distinct metabolic and hepatic profiles of non-obese and obese MASLD adolescents compared to healthy controls, emphasizing the potential utility of FibroScan parameters, FGF-21, and CK-18 as non-invasive tools in assessing MASLD severity. Elevated FGF-21 levels, particularly in obese MASLD, indicate its role as a marker of metabolic stress associated with liver dysfunction. FibroScan parameters effectively differentiated hepatic fat content and liver stiffness, reflecting the burden of hepatic steatosis and fibrosis risk. However, the cross-sectional design of this study limits definitive conclusions regarding causality or disease progression. Future longitudinal studies incorporating liver biopsy validation are necessary to establish these biomarkers’ predictive value and utility in monitoring disease progression and treatment response in pediatric MASLD populations.

## Figures and Tables

**Table 1.** Comparison of the Demographics Among the Groups

	**Control Group (n = 92)**		**Non-Obese MASLD (n = 32)**		**Obese MASLD (n = 39)**		***P***	***P*1**	***P*2**	***P*3**
	**Mean ± SD**	**Median (Min-Max)**	**Mean ± SD**	**Median (Min-Max)**	**Mean ± SD**	**Median (Min-Max)**				
Age, years	14.48 ± 2.63	14.75 (9-18)	13.76 ± 2.57	14.75 (9-17.9)	14.79 ± 2.22	15 (9.2-17.9)	.243	.182	.683	.078
Gender, n (%) Female Male	55 (59.8) 37 (40.2)		15 (46.9) 17 (53.1)		22 (56.4) 17 (43.6)		.447			
Puberty, n (%)	86 (93.5)		29 (90.6)		36 (92.3)		.864			
Weight, kg	50.93 ± 11.79	51.75 (24.5-77.6)	70.33 ± 13.21	51.75 (45-91.5)	94.5 ± 17.51	94 (58.4-138.3)	<.001*	<.001^¥^	<.001^¥^	<.001^¥^
Weight SD, kg	−0.21 ± 0.96	−0.28 (−2.23 to 2.17)	1.82 ± 0.85	−0.28 (0.22-3.34)	3.44 ± 1.05	3.32 (1.85-6.32)	<.001	<.001^¥^	<.001	<.001
Height, cm	158.53 ± 11.57	160 (130-180)	159.55 ± 11.23	160 (137.1-180)	163.76 ± 9.79	164.6 (138-184.9)	.043	.769	.012	.096^¥^
Height SD, cm	0.01 ± 0.97	0.06 (−2.06 to 2.56)	0.32 ± 1.13	0.06 (−2.37 to 2.74)	0.43 ± 1.29	0.24 (−2.64 to 3.74)	.088*	.131^¥^	.042^¥^	.715^¥^
BMI, kg/m^2^	19.91 ± 2.71	19.97 (14.5-26.3)	27.26 ± 2.35	19.97 (21.7-30)	35.03 ± 4.15	33.6 (30.4-45.2)	<.001	<.001	<.001	<.001
BMI SD, kg/m^2^	−0.25 ± 0.91	−0.34 (−2.01 to 1.65)	1.89 ± 0.6	−0.34 (0.1-3)	3.06 ± 0.52	2.96 (2.2-4.2)	<.001	<.001^¥^	<.001	<.001
SBP, mm Hg	109.84 ± 8.91	110 (90-125)	118.13 ± 8.87	110 (90-140)	120.13 ± 13.64	120 (100-160)	<.001	<.001	<.001	.976
SBP SD, mm Hg	0.18 ± 0.87	0.29 (−2.05 to 1.88)	0.93 ± 0.81	0.29 (−1.08 to 2.33)	0.84 ± 1	0.95 (−1.17 to 2.33)	<.001	<.001	.001	.711
DBP, mm Hg	69.18 ± 7.78	70 (50-80)	76.88 ± 6.32	70 (60-90)	75.38 ± 11.49	70 (60-100)	<001	<.001	.006	.514
DBP SD, mm Hg	0.5 ± 0.79	0.51 (−1.5 to 2.05)	1.28 ± 0.59	0.51 (−0.39 to 2.05)	0.9 ± 0.87	0.81 (−0.71 to 2.33)	<.001*	<.001^¥^	.011^¥^	.024^¥^
WC, cm	70.13 ± 7.75	70 (55-90)	94.06 ± 9.45	70 (76-115)	111.6 ± 13.07	109 (85-144)	<.001	<.001^¥^	<.001	<.001
Nigricans Acanthosis, n (%)	3 (3.3)		8 (25)		23 (59)		<.001			
Metabolic syndrome, n (%)	1 (1.1)		15 (46.9)		27 (69.2)		<.001			

BMI, body mass index; DBP, diastolic blood pressure; MASLD, metabolic dysfunction-associated steatotic liver disease; *P*, probability among the groups; *P*1, probability between the control group and non-obese non-alcoholic fatty liver disease patients; *P*2, probability between the control group and obese non-alcoholic fatty liver disease patients; *P*3, probability between non-obese non-alcoholic fatty liver disease patients and non-obese non-alcoholic fatty liver disease patients; SBP, systolic blood pressure; SD, standard deviation; WC, waist circumference.

^*^One-way ANOVA.

^¥^Student *t-*test.

**Table 2.** Comparison of the Laboratory Values Among the Groups

	**Control Group (n = 92)**		**Non-Obese MASLD (n = 32)**		**Obese MASLD (n = 39)**		***P***	***P*1**	***P*2**	***P*3**
	**Mean ± SD**	**Median ** **(Min-Max)**	**Mean ± SD**	**Median ** **(Min-Max)**	**Mean ± SD**	**Median ** **(Min-Max)**				
FBG, mg/dL	86.2 ± 7.89	86 (68-102)	88.41 ± 7.24	86 (75-101)	87.15 ± 7.38	87 (73-107)	.359*	0.166^¥^	.518^¥^	.475^¥^
Insulin, mIU/L	12.41 ± 6.03	12 (2.78-30)	28.71 ± 16.1	12 (9.9-93.7)	37.84 ± 22.26	33.8 (11.7-143.9)	<.001	<.001	<.001	.014
HOMA_IR	2.67 ± 1.42	2.57 (0.6-7.48)	6.31 ± 3.85	2.57 (2.17-22.45)	8.38 ± 5.33	7.26 (2.6-34.1)	<.001	<.001	<.001	.021
AST, IU/L	17.21 ± 4.15	16 (10-29)	24.49 ± 14.78	16 (8-78)	28.05 ± 14.52	28 (11-74)	<.001	.001	<.001	.158
ALT, IU/L	12.99 ± 7.05	11 (3-55)	42.19 ± 42.8	11 (11-167)	45.51 ± 35.41	34 (11-165)	<.001	<.001	<.001	.245
GGT, IU/L	11.13 ± 4.82	10 (3-37)	23.22 ± 17.25	10 (11-92)	24.95 ± 13.87	22 (7-75)	<.001	<.001	<.001	.179
ALP, IU/L	156.72 ± 104.54	108.5 (34-504)	177.03 ± 84.07	108.5 (39-329)	136.56 ± 71.64	108 (48-309)	.203	.151	.757	.057
Total cholesterol, mg/dL	143.04 ± 28.25	141 (81-211)	164.38 ± 35.96	141 (105-272)	160.9 ± 26.83	156 (114-218)	<.001*	.001^¥^	.001^¥^	.642^¥^
Triglycerides, mg/dL	79.21 ± 29.61	72.5 (30-184)	152.75 ± 66.74	72.5 (45-301)	153.41 ± 60.92	149 (71-318)	<.001	<.001	<.001	.822
LDL, mg/dL	75.41 ± 22.82	69.75 (30-154)	87.39 ± 28.57	69.75 (44.3-153)	87.8 ± 26.16	88 (48-161)	.015	.043	.01	.95^¥^
HDL, mg/dL	54.76 ± 13.13	54 (24-104)	45.91 ± 11.41	54 (27-80)	41.99 ± 8.64	42 (27-68)	<.001	<.001	<.001	.104^¥^
TSH, mIU/L	2.28 ± 1.12	2.06 (0.59-5.69)	3.08 ± 1.59	2.06 (0.86-7.35)	2.82 ± 1.48	2.7 (0.8-7.2)	.014	.008	.061	.467
fT4, ng/dL	1.22 ± 0.2	1.21 (0.91-1.93)	1.16 ± 0.14	1.21 (0.9-1.39)	1.14 ± 0.17	1.11 (0.82-1.66)	.037	.195	.015	.468^¥^
Vitamin D, mg/L	12.01 ± 6.05	11.23 (3.2-35.4)	11.81 ± 5.06	11.23 (6.02-29)	11.95 ± 7.44	9.8 (3-33)	.771	.993	.513	.521
Ca^2+^, mg/dL	9.38 ± 0.45	9.4 (7.56-10.3)	9.63 ± 0.41	9.4 (8.6-10.3)	9.56 ± 0.41	9.6 (8.2-10.3)	.008*	.007^¥^	.032^¥^	.495^¥^
P^+^, mg/dL	4.45 ± 0.73	4.4 (2.74-6.32)	4.43 ± 0.68	4.4 (2.8-6)	4.21 ± 0.6	4.1 (3.25-5.7)	.173*	.912^¥^	.07^¥^	.141^¥^
Uric acid, mg/dL	4.03 ± 0.95	3.9 (2-6.8)	5.33 ± 1.74	3.9 (2.4-8.6)	5.78 ± 1.64	5.6 (1.7-9)	<.001	<.001	<.001	.261^¥^
Ferritin, mg/L	29.35 ± 18.99	26 (5-98)	58.39 ± 36.52	26 (14-157)	50.19 ± 30.2	43 (9-141)	<.001	<.001	<.001	.481

ALP, alkaline phosphatase; ALT, alanine transaminase; AST, aspartate transaminase; fT4, free thyroxine; FBG, fasting blood glucose; HOMA-IR, homeostasis model assessment for insulin resistance; GGT, gamma-glutamyl transferase, HDL, high-density lipoprotein; LDL, low-density lipoprotein; *P*, probability among the groups; *P*1, probability between the control group and non-obese non-alcoholic fatty liver patients; *P*2, probability between the control group and obese non-alcoholic fatty liver patients; *P*3, probability between non-obese non-alcoholic fatty liver patients and non-obese non-alcoholic fatty liver patients; TSH, thyroid-stimulating hormone.

*One-way ANOVA.

^¥^Student *t*-test.

**Table 3.** Comparison of the Liver Stiffness Parameters Among the Groups

	**Control Group (n = 92)**		**Non-Obese MASLD (n = 32)**		**Obese MASLD (n = 39)**		***P***	***P*1**	***P*2**	***P*3**
	**Mean ± SD**	**Median (Min-Max)**	**Mean ± SD**	**Median (Min-Max)**	**Mean ± SD**	**Median (Min-Max)**				
CK-18, U/L	0.64 ± 0.77	0.48 (0.06-4.47)	0.78 ± 0.74	0.48 (0.06-3.63)	1.34 ± 2.77	0.56 (0.06-15.97)	.127	.202	.065	.644
FGF-21, pg/mL	7.31 ± 15.96	2.4 (0-128.6)	14.76 ± 24.74	2.4(0-127)	20.65 ± 36.1	8.6 (0-197.2)	.002	.031	.001	.38
FibroScan_CAP, dB/m	187.86 ± 23.98	191 (121-224)	283.03 ± 39.48	191 (233-380)	299.26 ± 47.45	296 (227-387)	<.001*	<.001^¥^	<.001^¥^	.127^¥^
FibroScan_LSM, kPa	4.15 ± 0.68	4.3 (2.3-6.1)	5.15 ± 1	4.3 (3.7-7.8)	6.03 ± 1.79	5.8 (3.8-11.9)	<.001	<.001^¥^	<.001	.042

CK-18, cytokeratin 18; FGF-21, fibroblast growth factor 21; *P*, probability among the groups; *P*1, probability between the control group and non-obese NAFLD patients; *P*2, probability between the control group and obese non-obese non-alcoholic fatty liver patients; *P*3, probability between non-obese non-obese non-alcoholic fatty liver patients and non-obese non-obese non-alcoholic fatty liver patients.

*One-way ANOVA.

^¥^Student *t*-test.

## Data Availability

The datasets used and/or analyzed during the current study are available from the corresponding author upon reasonable request.
